# Cardiorespiratory fitness and left ventricular recovery after kidney transplantation: evidence, gaps, and future directions

**DOI:** 10.1080/0886022X.2026.2626621

**Published:** 2026-03-02

**Authors:** Francesca E. Ridler, Matthew P. M. Graham-Brown, Roseanne E. Billany

**Affiliations:** a Leicester Medical School, College of Life Sciences, University of Leicester, United Kingdom; b Division of Cardiovascular Sciences, University of Leicester, Leicester, United Kingdom; c Leicester Partnership for Kidney Health Research, University of Leicester, Leicester, United Kingdom

**Keywords:** Cardiovascular disease, cardiorespiratory fitness, kidney transplant recipient, uremic cardiomyopathy, cardiac magnetic resonance imaging, peak oxygen uptake

## Abstract

**Background:**

Despite improved survival following kidney transplantation, cardiovascular disease (CVD) remains a leading cause of mortality in kidney transplant recipients (KTRs). This risk is driven by complex traditional and nontraditional mechanisms contributing to uremic cardiomyopathy. Cardiorespiratory fitness (CRF) is consistently reduced in KTRs and strongly associated with cardiovascular outcomes. However, while cardiac structure and function may partially improve post-transplant, recovery of CRF often remains incomplete compared to healthy individuals, suggesting that structural reverse remodeling does not necessarily equate to restored cardiovascular reserve.

**Methods:**

This review synthesises current evidence on post-transplant changes in left ventricular structure and function and trajectories of CRF recovery. We highlight persistent discrepancies between echocardiography-based and cardiac magnetic resonance (CMR)-based findings, together with the limited use of cardiopulmonary exercise testing (CPET) in longitudinal studies.

**Key findings:**

We discuss the concept of a ‘transplant cardio-recovery gap’, reflecting the dissociation between structural normalisation and functional capacity restoration.

**Future directions:**

We outline future directions for research including phenotype-specific monitoring using CMR-derived strain, native T1 mapping, and CPET parameters, integrated through AI-enabled predictive analytics, to enable digital twin models capable of forecasting individualised recovery trajectories. We discuss CMR–CRF coupling models, and adaptive rehabilitation trials stratified by functional cardiovascular reserve rather than structural metrics alone.

**Conclusion:**

While kidney transplantation offers partial cardiovascular recovery, restoration of cardiopulmonary resilience remains an unmet therapeutic target. Precision, AI-guided CRF evaluation and rehabilitation may redefine cardiovascular risk management in KTRs and inform the next generation of transplant optimisation strategies.

## KEY TEACHING POINTS

1. Kidney transplant recipients experience persistent cardiovascular risk post-transplant.
2. There is limited and heterogeneous left ventricular recovery.
3. Cardiorespiratory fitness improves but rarely normalises.
4. There is weak evidence linking cardiorespiratory fitness and left ventricular remodeling.
5. Improvements in left ventricular structure and function do not fully translate to significant improvement in cardiorespiratory fitness.
6. There is a need for multimodal, AI-enabled, longitudinal research.

## Introduction

This narrative literature review aims to describe existing evidence on structural and functional changes in the left ventricle of kidney transplant recipients (KTRs), emphasizing inconsistencies in reported cardiac remodeling, as well as highlighting the low levels of cardiorespiratory fitness (CRF) in this population. Research that has directly investigated possible relationships between changes in left ventricular (LV) structure and function and CRF is reviewed. Notably, improvements in LV structure and function after transplant do not fully translate to restored cardiovascular reserve, which has been termed the ‘transplant cardio-recovery gap’. As well as summarising current evidence, this work aims to propose future models, phenotypes, and trial frameworks, suggesting a role for artificial intelligence (AI)-driven modeling with digital twin phenotyping to allow for precision rehabilitation.

Cardiovascular disease (CVD) is a leading cause of death in patients with chronic kidney disease (CKD) [1]. Although kidney transplantation is considered the gold standard treatment for end-stage kidney disease (ESKD) [2], KTRs still have a reduced life expectancy compared to the general population [3] with CVD remaining a primary contributor to this mortality [4]. Common types of CVD in KTRs include coronary artery disease, heart failure, cardiac arrhythmias and structural heart disease [5]. Kidney transplant recipients experience higher rates of myocardial infarction, stroke and heart failure compared to the general population [6] and CVD is often the cause of readmission to hospital post-transplant [7]. Many CVD-related deaths in KTRs are sudden, due to the high prevalence of structural as well as atherosclerotic heart disease [8].

This increased CVD risk is due to a complex combination of traditional and nontraditional risk factors that cluster together to drive pathological cardiovascular structural and functional changes that are collectively termed uremic cardiomyopathy [9], a condition characterised by left ventricular hypertrophy (LVH), myocardial fibrosis and left ventricular cavity dilation, leading to systolic and diastolic dysfunction [10]. Uremic cardiomyopathy is diagnosed using cardiovascular imaging, typically echocardiography and cardiac magnetic resonance imaging (CMR) [9]. Cardiac MRI is considered the gold standard method of cardiovascular imaging [11] due to its accuracy, detailed soft tissue characterisation and high reproducibility [11,12]. However, echocardiography is more commonly used [13], as it is widely available, minimally invasive, low cost and portable in nature [12].

Cardiac assessment frequently prioritises the structure and function of the left ventricle [14] due to its central role in maintaining systemic circulation and its relevance in a wide range of cardiovascular conditions. As the primary chamber responsible for generating sufficient cardiac output to perfuse vital organs [15], the left ventricle is fundamental to overall health. The left ventricle functions under high pressure [15], making it particularly susceptible to pathological changes such as hypertrophy. Consequently, this review will focus specifically on the structural and function changes in the left ventricle.

A common risk factor for CVD in KTRs is reduced cardiorespiratory fitness [16]. Cardiorespiratory fitness is defined as the ability of the cardiovascular and respiratory systems to supply the mitochondria of skeletal muscle with oxygen during sustained physical activity [17]. Patients with CKD often have significantly lower CRF than the general population, estimated at 50-80% of that of healthy individuals [18]. Poor CRF has been shown to associate with a greater mortality risk in patients on hemodialysis during a median 39-month follow-up period [19], and with post-transplant mortality in KTRs [20]. However, research on CRF in KTRs is limited.

The gold standard method of measuring CRF is cardiopulmonary exercise testing (CPET) [21]. The test provides information on the functioning of the cardiovascular, respiratory and skeletal muscle systems during exercise, and involves directing patients to exercise at a gradually increasing intensity until they reach their peak capacity, usually on a cycle ergometer or a treadmill [22]. Exploring relationships between CRF and cardiovascular structure and function in KTRs is important to develop strategies to address the high levels of cardiovascular morbidity and mortality in this patient group.

## Left ventricular remodeling in kidney transplant recipients: partial, transient, or absent?

The left ventricle of the heart is responsible for pumping oxygenated blood through the aortic valve to maintain circulation to the other organ systems of the body [15]. Consequently, dysfunction results in significant disease symptoms [15]. Patients with ESKD are prone to LV dysfunction due to traditional and nontraditional risk factors, which has been associated with morbidity and mortality [9]. Key parameters of LV structure and function are summarised in Table 1, alongside their associated clinical outcomes. Study characteristics for the studies discussed are summarised in Table 2.

**Table 1.** Definition of each left ventricular parameter with their associated outcomes.

LV parameter (unit)	Explanation	Associated outcomes
LVMi (g/m^2^)	The mass of the left ventricle adjusted for body size.	Increased LVMi is associated with increased mortality risk and cardiovascular events, including sudden cardiac death.
LV volume index (mL/m^2^)	The volume of the left ventricle adjusted for body size.	Dilation of the left ventricle is linked to cardiovascular mortality in ESKD.
LV diameter (mm)	The diameter of the left ventricle.	Dilation of the left ventricle is linked to cardiovascular mortality in ESKD.
LVEF (%)	The fraction of blood ejected from the left ventricle during systole.	Low LVEF is strongly associated with cardiac mortality.
Myocardial strain (GCS, GRS, GLS) (%)	The change in myocardial fiber length in the circumferential, radial or longitudinal direction during systole. It is a more sensitive measure of systolic dysfunction that can detect subclinical changes before an overt reduction in LVEF is present.	Worsening strain has been associated with adverse cardiac events.
Native T1 time (ms)	Relaxation time of the myocardium. Increased T1 times typically represent myocardial fibrosis, though not exclusively.	Increased T1 time has been associated with cardiac events and mortality.

*Abbreviations:* LVMi: left ventricular mass index, LV: left ventricle, LVEF: left ventricular ejection fraction, GCS: global circumferential strain, GRS: global radial strain, GLS: global longitudinal strain.

**Table 2.** Study characteristics for studies exploring cardiac structure and function in kidney transplant recipients.

Author Year	Study type	Sample size and population	Follow up time	Imaging modality	Summary of main outcomes*
Kensinger et al. 2017 [24]	Prospective cohort	143 KTRs	24 months	Echo	↓LVMi ↑LVEF
Hawwa et al. 2015 [25]	Retrospective cohort	232 KTRs	Between 12 and 24 months	Echo	↓LVMi ↓LVDD ↑LVEF
An et al. 2015 [26]	Retrospective cohort	767 KTRs	7.5 years	Echo	↓LVMi ↓LVESD ↓LVEDD ↑LVEF
Tian et al. 2024 [27]	Systematic review and meta-analysis	4122 (46 studies) KTRs	N/A	Echo, CMR	No difference LVMi reduction between KTRs and patients on dialysis
Barbosa et al. 2021 [28]	Prospective cohort	44 KTRs 10 age- and sex-matched controls	6 months	CMR	↔LVMi ↔LVEDV ↓T1 time ↔LVEF ↑ GLS ↔ GRS ↔ GCS
Prasad et al. 2018 [29]	Prospective cohort	39 KTRs 43 transplant candidates on dialysis	12 months	CMR	↔LVMi ↓LVESVi ↓LVEDVi ↔LVEF
Patel et al. 2008 [30]	Prospective cohort	25 KTRs 25 transplant candidates	2.8 years for KTRs and 2.4 years for KT candidates	CMR	↔LVMi ↔LVSD ↔LVEF
Akkaya et al. 2024 [31]	Retrospective cohort	145 KTRs	At least 2 years	Echo	↔LVH prevalence
Panisset et al. 2024 [32]	Retrospective cohort	80 KTRs	39 months	Echo	↔LVMi ↔LVEDVi
Qi et al. 2022 [33]	Retrospective cohort	53 KTRs 21 age and sex-matched controls	3, 6 and 9 months	CMR	↓LVEDVi ↓LVESVi ↑LVEF ↑GRS ↑GCS ↓Native T1 times
Hayer et al. 2019 [34]	Prospective cohort	24 KTRs 18 age- and sex-matched controls	2 months	CMR	↔LVESVi ↓LVEDVi ↑LVEF ↔ GLS ↑Native T1 times
Gong et al. 2018 [35]	Prospective cohort	39 KTRs 40 ESKD on dialysis	12 months	CMR	↔LVESVi ↔LVEDVi ↑LVEF but no difference when compared to dialysis patients ↓GLS ↑GRS ↑GCS
Shimony et al. 2019 [38]	Retrospective cohort	113 KTRs	Approximately 510 days	Echo	↓LVESD ↓LVEDD But only when eGFR< 45
Pickup et al. 2021 [36]	Systematic review and meta-analysis	1998 (26 studies) KTRs	1 week to 5 years	Echo, CMR	↓LVIDD ↔LVEDV when analyzing CMR-based studied ↑LVEF overall, but no significant difference compared to dialysis patients.
Kim et al. 2019 [37]	Retrospective cohort	97 KTRs with normal preoperative systolic function	Average of 18.9 months	Echo	↓LVDD
d’Hervé et al. 2024 [39]	Retrospective cohort	106 KTRs	Average of 22 months	Echo	↔LVEDVi ↔LVEF
Contti et al. 2019 [40]	Prospective cohort	44 KTRs	6 months	CMR	↔LVEDV ↔LVSD
Skalsky et al. 2024 [41]	Retrospective cohort	604 (293 for final analysis) KTRs	Up to 2 years	Echo	↑LVEF
Xu et al. 2017 [42]	Retrospective cohort	72 KTRs	Average of 228 days	Echo	↔LVEF
Kim et al. 2023 [43]	Retrospective cohort	488 KTRs	Within 3 years	Echo	↑GLS

* Unless otherwise stated, all outcomes are compared to pre-transplant levels.
↑ Increase ↓ Decrease ↔ No significant change

*Abbreviations:* KTR: kidney transplant recipient; LVMi: left ventricular mass index; LVESVi: left ventricular end-systolic volume index; LVEDVi: Left ventricular end-diastolic volume index; LVESD: left ventricular end-systolic diameter; LVEDD: left ventricular end-diastolic diameter; LVIDD: left ventricular internal diastolic diameter; LVEF: left ventricular ejection fraction; GLS: global longitudinal strain; GRS: global radial strain; GCS: global circumferential strain; LVH: left ventricular hypertrophy.

## Left ventricular mass

Numerous studies have reported regression of LV mass index (LVMi) after kidney transplantation, suggesting transplantation may lead to beneficial structural and functional changes in the heart that restore a more normal state, known as reverse remodeling [23]. In a prospective cohort study of 143 KTRs assessing echocardiographic changes at one-, 12-, and 24-months post-transplant, Kensinger et al. reported a reduction in LVMi of 10.82 g/m^2^ (*p* < 0.001) between one month and 12 months, with an additional decrease of 4.77 g/m^2^ (*p* < 0.001) by month 24, after adjustment for potential confounders [24]. Overall, 17% of participants with elevated LVMi at one month achieved normalisation by 12 months. However, an additional 9% with normal baseline LVMi developed *de novo* LVH by the same time point, and relative wall thickness remained unchanged. Furthermore, this study was limited by significant loss to follow-up, with only 85 participants remaining at 12 months and 33 at 24 months.

Similarly, in a retrospective cohort study of 232 participants comparing pre-transplant echocardiograms to those performed at a median of 422 days post-transplant, Hawwa et al. demonstrated an overall reduction in LVMi of 7 g/m^2^ (*p* = 0.032) [25]. Those with baseline left ventricular systolic dysfunction and severe dysfunction showed more pronounced reductions of 20 g/m^2^ (*p* < 0.0001) and 38 g/m^2^ (*p* < 0.0001), respectively. This study also reported a 0.1 cm decrease in both interventricular septal (*p* < 0.001) and posterior wall diameters (*p* < 0.0001) in individuals with a baseline diameter exceeding 1.2 cm. Longer term improvements in cardiac structure were demonstrated in a large retrospective study of 767 KTRs, which reported reductions in LVMi and posterior wall thickness (PWT) at both one- and five-years post-transplant (*p* < 0.05) [26]. Interventricular septal diameter (IVSD) reduction only became significant at five years, and age-normalised relative wall thickness (RWT) remained unchanged at both time points.

However, the most recent systematic review on LVH in KTRs challenges these findings. This review analysed 46 studies, encompassing a total of 4122 participants, and while it did report a significant decrease in LVMi in KTRs after transplant (standard mean difference −0.44 g/m^2^, 95% CI: −0.60 to −0.28), when the data was subdivided based on imaging technique it reported that LVMi measured with CMR did not improve significantly [27]. Three of the five studies in the review that used CMR reported no improvement in LVMi at six months [28], 12 months [29], or 2.8 years [30] post-transplant. Additionally, the change in LVMi in KTRs after transplant was found to not be significantly different from the change in LVMi in dialysis patients after a similar follow-up time. The review emphasised the poor quality of existing evidence due to lack of randomisation, limited control groups, and small sample sizes, as well as highlighting the predominance of echocardiographic data over CMR.

Additional echocardiography-based studies published after this systematic review also support a limited effect of kidney transplantation on improving LVMi. Akkaya et al. analysed echocardiograms from 145 KTRs, comparing pre-transplant measurements with those taken at least two years after transplant. The study found no significant changes in LVH prevalence or interventricular septal diameter over time [31]. A further study examining post-transplant echocardiograms taken at an average of 17 and 39 months also found no significant alteration in LVMi [32].

## Left ventricular dimensions

Multiple CMR-based studies have demonstrated reductions in left ventricular volumes following kidney transplantation. Qi et al. observed significant decreases in left ventricular end-diastolic volume index (LVEDVi) and end-systolic volume index (LVESVi) at three-, six-, and nine-months after transplant, with reductions of 13.74 mL/m^2^ (*p* = 0.02) and 12.17 mL/m^2^ (*p* < 0.001) at nine months, respectively [33]. Hayer et al. reported a 16 mL/m^2^ reduction in LVEDVi at two months after transplant (*p* < 0.05), though LVESVi did not significantly change and the small sample size (*n* = 24) may limit generalisability [34]. Prasad et al. found that reductions in LVEDVi and LVESVi in KTRs 12 months after transplant were significantly greater than the reduction seen in dialysis patients after the same time period (−4.9 ± 8.5 mL/m^2^ vs 0.3 ± 9.2 mL/m^2^, *p* = 0.022; −3.36 ± 5.6 mL/m^2^ vs −0.22 ± 4.4 mL/m^2^, *p* = 0.009, respectively) [29]. Similarly, Gong et al. reported no volume changes in dialysis patients after 12 months follow up, while KTRs showed a decline of 12 mL/m^2^ in LVEDVi and 7 mL/m^2^ in LVESVi, although the KTRs had higher baseline values [35].

There is also evidence to suggest that kidney transplantation improves left ventricular diastolic and systolic diameters as well as volumes, although the majority is based on echocardiographic data. Pickup et al.’s systematic review on LV structural and functional changes associated with kidney transplantation reported that eight out of 13 echocardiographic studies found a significant decrease in left ventricular internal diastolic diameter (LVIDD). However, it did highlight high levels of heterogeneity and low quality of evidence in existing studies [36]. Additionally, a large retrospective cohort study of 767 KTRs reported a slight decrease of 1 mm (*p* < 0.05) in both left ventricular end-diastolic diameter (LVEDD) and end-systolic diameter (LVESD) at one year after transplant [26]. In similar retrospective studies, Hawwa et al. [25] and Kim et al. [37] and reported left ventricular diastolic diameter (LVDD) reductions of 1 mm (*p* = 0.005) at an average of 422 days after transplant and 4 mm (*p* < 0.001) at an average of 22.4 months after transplant, respectively. Both studies reported greater decreases in those with baseline LVDD >5.6 cm, suggesting those with pre-transplant cavity enlargement show the most improvement. Shimony et al. found decreases in LVEDD (−2.8 mm, *p* = 0.009) and LVESD (−2.3 mm, *p* = 0.03) in patients with low eGFR, with the trend losing significance when eGFR exceeded 45 mL/min/1.73m^2^ [38].

In contrast, some evidence suggests that kidney transplantation may have a limited impact on left ventricular volumes and diameters. Pickup et al. conducted a meta-analysis of CMR-based studies exploring LV structural changes in KTRs after transplant and reported no significant change in LVEDV [36]. Whilst this analysis was based on only two studies, the results are consistent with two larger echocardiographic retrospective cohort studies that both reported no change in LVEDVi, one at an average of 22 months after transplant [39], and one comparing post-transplant scans at an average of 17 months and 39 months [32]. An additional two CMR-based studies also showed no change in LVEDV post-transplantation [28,40]. Furthermore, CMR-based studies by Contti et al. [40] and Patel et al. [30] demonstrated no change in left ventricular systolic diameter (LVSD) at six months and an average of 2.8 years, respectively.

## Left ventricular ejection fraction

The majority of studies suggest transplantation has a positive impact on LVEF. A retrospective cohort study of 293 participants found that 28% of KTRs experienced at least a 5% improvement in LVEF within two years after transplant, a figure that increased to 52% among those with a baseline LVEF below 40% [41]. These findings align with those of Hawwa et al. who reported an overall 3% increase (*p* = 0.002) in LVEF at an average of 422 days post-transplant, with greater improvements observed in patients with baseline left ventricular systolic dysfunction (9%, *p* < 0.0001) and moderate left ventricular systolic dysfunction (15%, *p* < 0.0001) [25]. Kensinger et al. observed a gradual improvement in LVEF after transplant, with increases of 1.54% (*p* = 0.039) at 12 months and 2.62% (*p* = 0.008) at 24 months after transplant [24]. An et al. demonstrated long-term improvements in LVEF at one year and five years, although these increases were small at 1% and 1.7%, respectively (*p* < 0.05) [26]. Additionally, two CMR-based studies also reported improvements in LVEF after transplant. Qi et al. reported an 8.45% (*p* = 0.002) improvement nine months after transplant [33], while Hayer et al. reported a 5% (*p* < 0.05) increase over two months [34]. These results suggest that transplantation may be beneficial in certain patients with established systolic heart failure.

However, several CMR-based studies contradict these findings and instead suggest minimal improvement in LVEF following kidney transplantation. A 2021 systematic review of 26 studies involving 1998 participants reported an overall increase in LVEF after transplant, but noted that the three studies that used CMR [29,30,34] found no significant difference between the change in LVEF in KTRs and the change in control groups [36]. Of these three studies, Prasad et al. observed no improvement in LVEF at 12 months in either KTRs or dialysis patients [29], while Patel et al. reported the same result at an average of 2.8 years after transplant [30]. Several additional CMR studies further support these findings. Gong et al. compared 39 KTRs with 40 dialysis patients and found that although LVEF significantly improved at 12 months after transplant in KTRs, the change was not statistically significant when compared to the change in dialysis patients after the same follow-up time [35]. Additionally, Barbosa et al. found no significant change in LVEF at six months after transplant [28].

Further supporting these findings, some echocardiographic data also indicates a lack of improvement in LVEF. d’Herve et al. reported no significant change at an average of 22 months after transplant in a cohort of 106 participants [39], while Xu et al. found no improvement at three months in a study of 72 participants [42]. However, the short follow-up period in the latter study raises the possibility that systolic dysfunction may have developed later.

## Myocardial strain

There is evidence to suggest that kidney transplantation improves myocardial strain. A large study using two-dimensional speckle-tracking echocardiography retrospectively studied 488 dialysis patients with impaired baseline global longitudinal strain (GLS) and found significant post-transplant improvements across all severity levels. The greatest improvement was found in those with severe impairment at baseline (4.9%, *p* < 0.001) [43]. Additionally, a CMR-based study of 53 KTRs showed a trend toward improvement in GLS at 14 months after transplant, which did not reach statistical significance but did reach levels comparable to healthy controls. The study also reported significant improvements in global radial strain (GRS) and global circumferential strain (GCS) at six months (*p* = 0.02, *p* = 0.03, respectively) [33].

However, evidence is not consistent. Gong et al. found GRS and GCS improved over 12 months (*p* = 0.003, *p* = 0.007, respectively) compared to a group of dialysis patients who showed no change in strain, but found that GLS worsened after 12 months in the KTR group (*p* = 0.026) [35]. Another study of 44 KTRs reported a modest but significant improvement in GLS at six months (1.8%, *p* < 0.001), though still below healthy control levels, and no changes in GRS or GCS [28]. Hayer et al. reported no significant change in GLS at two months after transplant [34].

## Myocardial fibrosis and edema

Some small studies have suggested that kidney transplantation leads to improvements in native T1 and T2 times. In a retrospective cohort study of 53 KTRs, Qi et al. reported that global left ventricular native T1 times decreased as early as three months following transplantation (1229.32 ms ±40.18 at baseline vs 1170.61 ms ±47.41, *p* = 0.002), suggesting transplantation has a beneficial effect on myocardial fibrosis [33]. In an additional small prospective study of 44 KTRs comparing CMR images at ten days and six months post-transplant, Barbosa et al. also reported a decrease in mean native T1 times (1331 ± 52 ms vs 1298 ± 42 ms, *p* < 0.001), although it remained higher than healthy controls at both time points [28].

In contrast, Hayer et al. reported an increase in septal native T1 times (985 ± 25  ms  vs. 1002 ± 30 ms, *p* = 0.014) and no change in global native T1 times at two months post-transplant [34]. However, this study was limited by its small sample size of 24 KTRs and 18 controls.

In conclusion, the current literature presents mixed results regarding the impact of kidney transplantation on cardiovascular structure and function in KTRs. While several studies suggest improvements post-transplantation, particularly in patients with a level of baseline dysfunction, these findings are often limited by small sample sizes and short follow-up periods. Notably, evidence from CMR-based studies frequently fails to corroborate echocardiographic findings, which raises questions about the extent of reverse remodeling post-transplant. The findings discussed indicate that although kidney transplantation may result in cardiovascular benefits for some patients, its effects are not consistent and high-quality, longitudinal research is needed to clarify the extent of cardiac recovery in this population.

## Cardiorespiratory fitness in kidney transplant recipients: the transplant cardio-recovery gap

Patients with CKD have reduced levels of CRF compared to the general population [18]. The following research suggests that, while KTRs typically perform better than controls with kidney failure, they still exhibit low levels of CRF compared to healthy individuals. This poor CRF has been associated with poor post-transplant outcomes in KTRs [20]. Low CRF in KTRs despite evidence to suggest structural and function cardiovascular remodeling highlights the need to address the ‘transplant cardio-recovery gap’, which we define as the asynchronous recovery of left ventricular function and CRF after transplant.

The gold standard test for CRF is CPET [21]. Relevant CPET parameters and their associated clinical outcomes have been summarised in Table 3. Study characteristics for the studies discussed are summarised in Table 4.

**Table 3.** Definition of each CPET parameter with their associated outcomes.

CPET parameter (unit)	Explanation	Associated outcomes
V̇O_2 max_ (L/min)	The maximum possible rate that the body can transport and use oxygen during exertion. In practice, determining true V̇O_2 max_ is difficult to achieve, especially in older and frailer patients, so V̇O_2 peak_ is more commonly reported.	Low V̇O_2 max_ has been associated with mortality in ESKD patients as well as in other patient groups including chronic heart failure and diabetes.
V̇O_2 peak_ (L/min)	The rate of oxygen transport at the greatest physical exertion that was achieved during CPET.	As above
V̇O_2 peak_ (mL/kg/min)	The rate of oxygen transport and utilisation at the greatest physical exertion that was achieved during CPET, adjusted for body size.	As above
V̇O_2_AT (mL/kg/min)	The rate of oxygen uptake at the anaerobic threshold, which is the point at which there is a disproportionate rise in V̇E (volume of expired air [or inspired air] per minute) and V̇CO_2_ (volume of expired carbon dioxide) compared to V̇O_2_ and indicates the point at which activities cannot be sustained for long periods of time.	Low V̇O_2_AT has been associated with increased CVD and all-cause mortality risk in the general population, with values of <11 mL/kg/min widely accepted as a cutoff for post-surgical complications.
Maximum heart rate	Maximum heart rate achieved during CPET.	Inability to meet age-predicted maximum heart rate is a predictor of cardiovascular events and mortality.
Workload (W)	The power exerted by the participants during CPET performed on a cycle ergometer.	Prognostic data on workload in KTRs is limited, but maximal workload indexed to body weight has been associated with hospitalisation and death in other populations, such as patients with heart failure.
V̇EV̇CO_2_ slope	The relationship between ventilation and CO_2_ production, with a higher value suggesting less efficient ventilation as more effort is required to expire the same amount of CO_2_.	Prognostic data is limited in KTRs but studies in patients with heart failure have shown that V̇EV̇CO_2_ is associated with cardiac events and mortality risk.

*Abbreviations: V̇O_2 peak_*: peak oxygen uptake, *V̇O_2_AT:* oxygen uptake at the anaerobic threshold, *Max HR:* maximum heart rate, *V̇EV̇CO_2 min_*: ventilation to expired carbon dioxide ratio.

**Table 4.** Study characteristics for studies exploring cardiorespiratory fitness in kidney transplant recipients.

Author year	Study type	Sample size and population	Follow up time	CPET type	Summary of main outcomes*
Lim et al. 2020 [44]	Prospective cohort	81 KTRs 85 CKD stage 5 87 Hypertensive controls	1 year	Cycle ergometer	↑V̇O_2 peak_
Pella et al. 2023 [45]	Systematic review and meta analysis	207 (6 studies) KTRs	N/A	Not stated	↑V̇O_2 peak_ ↑V̇O_2_AT ↔Max HR
Theodorakopoulou et al. 2021 [46]	Systematic review and meta analysis	12 studies in the systematic review. 461 (8 studies) in the meta analysis KTRs and patients with kidney failure	N/A	Not stated	↑ V̇O_2 peak_ ↑ V̇O_2_AT compared to kidney failure patients
Painter et al. 2011 [47]	Prospective cohort	20 KTRs 23 dialysis patients 34 healthy controls	6 months	Treadmill	↑V̇O_2 peak_
Trájer et al. 2015 [48]	Cross-sectional	21 athlete KTRs 4 athlete liver transplant patients	N/A	Treadmill	V̇O_2 peak_ exceeded age and sex predicted values
Habedank et al. 2009 [49]	Prospective cohort	25 KTRs	12 months	Treadmill	↓V̇O_2 peak_ initially, but returned to baseline at 1 year ↓V̇EV̇CO_2_
Peterson et al. 2011 [50]	Cross-sectional	9 KTRs 10 HD 10 healthy controls	N/A	Cycle ergometer	No difference in V̇O_2_ peak, max HR or max WL between KTRs and dialysis patients
van den Ham et al. 2005 [51]	Cross-sectional	35 KTRs 16 HD 21 healthy controls	N/A	Cycle ergometer	No difference in V̇O_2 peak_ or maximum WL between KTRs and HD, but both lower than controls
Angell et al. 2021 [52]	Prospective cohort	24 KTRs 28 Kidney donors	14 weeks	Cycle ergometer	↑ V̇O_2_AT ↑ max HR
Patti et al. 2021 [53]	Cross-sectional	21 KTRs at 3 months post-op 14 KTRs at 12 months post-op 16 healthy controls	N/A	Cycle ergometer	Higher max HR and max WL in the 12-month group compared to the 3-month group Lower V̇EV̇CO_2_ in the 12-month group compared to the 3-month group
Armstrong et al. 2006 [54]	Cross-sectional	71 KTRs 25 healthy age- and sex-matched controls	N/A	Treadmill	61% achieved target HR
Schneider et al. 2020 [55]	Cross-sectional	12 KTRs 20 hemodialysis patients 26 pre-dialysis	N/A	Treadmill	↔ max HR compared to dialysis patients
Hellman et al. 2022 [57]	Prospective cohort	43 KTRs 50 dialysis 29 no dialysis	Within 3 years	Cycle ergometer	↓ Wlast4

* Unless otherwise stated, all outcomes are compared to pre-transplant levels.

*Abbreviations:* KTR: kidney transplant recipient; HD: hemodialysis; V̇O_2 peak_: peak oxygen uptake; V̇O_2_AT: oxygen uptake at the anaerobic threshold; Max HR: maximum heart rate; max WL: maximum workload; V̇EV̇CO_2 min_: ventilation to expired carbon dioxide ratio; Wlast4: workload in the last four minutes of peak exertion.

## Peak oxygen uptake

The largest study to date on this subject is a prospective cohort study by Lim et al. who measured CRF in 81 KTRs at baseline and up to one year after transplant, as well as in 85 transplant candidates and 87 hypertensive controls without CKD at the same time points [44]. The study reported a significant improvement in V̇O_2 peak_ in KTRs at 12 months after transplant (22.5 ± 6.3 mL/min^−1^/kg^−1^, *p* < 0.001), while transplant candidates experienced a decline over the same period. However, despite improving from baseline, the V̇O_2 peak_ in the KTRs remained lower than in the hypertensive controls at 12 months after transplant. These findings must be interpreted with caution as the controls were approximately ten years older than the KTRs, as age plays an important role in CRF.

A recent systematic review and meta-analysis on the effect of kidney transplantation on CRF supports these findings [45]. The review analysed six studies (207 participants) comparing measures of CRF in KTRs before and after transplant and reported a marginal increase in V̇O_2 peak_ (standardised mean difference: 0.50, 95% CI: 0.03, 0.98). However, this increase only became statistically significant after the removal of two low quality articles and only became evident after three months. An older systematic review of eight articles (461 participants) that compared CRF in KTRs to CRF in patients with kidney failure reported that KTRs had significantly higher V̇O_2 peak_ than the patients on dialysis (standardised mean difference: 0.70, *p* = 0.002) [46]. Additionally, it also reported significant improvement in V̇O_2 peak_ in KTRs after transplant compared with pre-transplant levels (weighted mean difference: 2.43 mL/kg/min, *p* = 0.02).

Additionally, a prospective study by Painter et al. demonstrated an increase in V̇O_2 peak_ six months after transplant (adjusted mean change: +0.28 L/min, *p* < 0.01) which was attributed to an increased cardiac output through increased heart rate but no change in stroke volume [47]. However, despite improvements, they only reached 79% of their age-predicted V̇O_2 peak_. Interestingly, a study of the Hungarian national transplant team demonstrated that the athletes exceeded their age-predicted V̇O_2 peak_ which, while this cohort is not generalisable to the broader KTR population, does demonstrate that it is possible to achieve normal levels of CRF after a kidney transplant [48].

In contrast, a few small studies have shown that transplantation has a limited impact on V̇O_2 peak_. In a prospective study of 25 participants, Habedank et al. found that V̇O_2 peak_ levels declined significantly in the early stages after transplant and took one year to return to baseline [49]. Additionally, two small cross-sectional studies that both compared CRF in KTRs to CRF in a group of patients on dialysis reported no difference in V̇O_2 peak_ between the two groups [50,51].

## Oxygen uptake at the anaerobic threshold

Kidney transplantation appears to lead to significant improvements in V̇O_2_AT compared to pre-transplant levels. In a systematic review of six studies (207 KTRs) Pella et al. demonstrated a significant improvement in V̇O_2_AT after kidney transplantation compared with pre-transplant levels (2.30 mL/kg/min, 95% CI 0.50, 4.09), although this was only based on two studies [45]. A small prospective study by Angell et al. that compared the change in V̇O_2_AT before and after transplant in 24 KTRs to the change in V̇O_2_AT in 28 kidney donors over the same time period demonstrated that V̇O_2_AT not only improved after transplantation but by the 14th week had reached a similar level to that of the kidney donors at baseline [52]. Furthermore, an older systematic review by Theodorakopoulou et al. that compared CRF in KTRs to CRF in patients with kidney failure reported that V̇O_2_AT was significantly higher in the KTRs (weighted mean difference: 3.14 mL/kg/min, 95% CI 2.39, 3.90). However, this was also only based on two studies [46].

## Heart rate

Several studies have shown that kidney transplantation is associated with an improved maximum heart rate. In a small cross-sectional study, Patti et al. demonstrated that peak heart rate was higher in KTRs that were 12 months post-transplant compared to KTRs that were three months post-transplant (144 bpm ± 21.41 vs 133 bpm ± 20.56, *p* < 0.05) [53]. However, the KTRs that were 12 months post-transplant still had a significantly lower maximum heart rate than that of a group of healthy controls. Angell et al. showed that maximum heart rate increased by the 7th week after surgery (*p* < 0.005), although it remained the same at week 14 [52]. In a cross-sectional study of 71 KTRs, 61% achieved their target heart rate [54].

On the other hand, a systematic review by Pella et al. showed no significant difference in maximum heart rate after transplantation compared to baseline values [45]. Furthermore, two small cross-sectional studies showed no difference in peak heart rate between KTRs and patients on dialysis [51,55].

## Workload

The evidence for the effect of kidney transplantation on maximal workload is limited. A small cross-sectional study conducted in 2005 showed that workload at peak exertion was higher in KTRs than in patients on hemodialysis (132 W ± 51 vs 113 W ± 52), but was still lower than in healthy controls [51]. A similar cross-sectional study published in 2021 reported that maximal power output was higher in a group of KTRs 12 months post-transplant compared to a group of KTRs three months post-transplant (70 *W* ± 33.29 vs 51 *W* ± 15.70), but was still lower compared to a group of healthy controls [53]. Although limited, these findings suggest transplantation may have a beneficial impact on workload achieved by patients. However, a larger prospective study contradicts these results, reporting that the mean workload in the last four minutes of maximal stress during CPET decreased in KTRs at three years post-transplant compared to pre-transplant levels (106 W ± 35 vs 96 W ± 33, *p* = 0.005).

## Ventilatory efficiency (V̇EV̇CO_2_ slope)

A small body of evidence suggests ventilatory efficiency may improve after kidney transplantation. Habedank et al. showed that despite initially worsening V̇EV̇CO_2_ after transplantation, values returned to baseline by three months and by 12 months had improved beyond baseline (28.7 ± 3.3, *p* = 0.03) [49]. However, this was a small study of only 25 Caucasian patients. Patti et al. reported that V̇EV̇CO_2_ was lower in a group of participants 12 months after transplant compared to a group three months after transplant, although the difference did not reach statistical significance [53]. However, the three-month group had significantly higher levels than a group of healthy controls (24.91 ± 2.93 vs 29.28 ± 4.25, *p* < 0.05), whereas the 12-month group showed no statistical difference to the controls, suggesting better ventilatory efficiency in the 12-month group.

**Figure 1.**
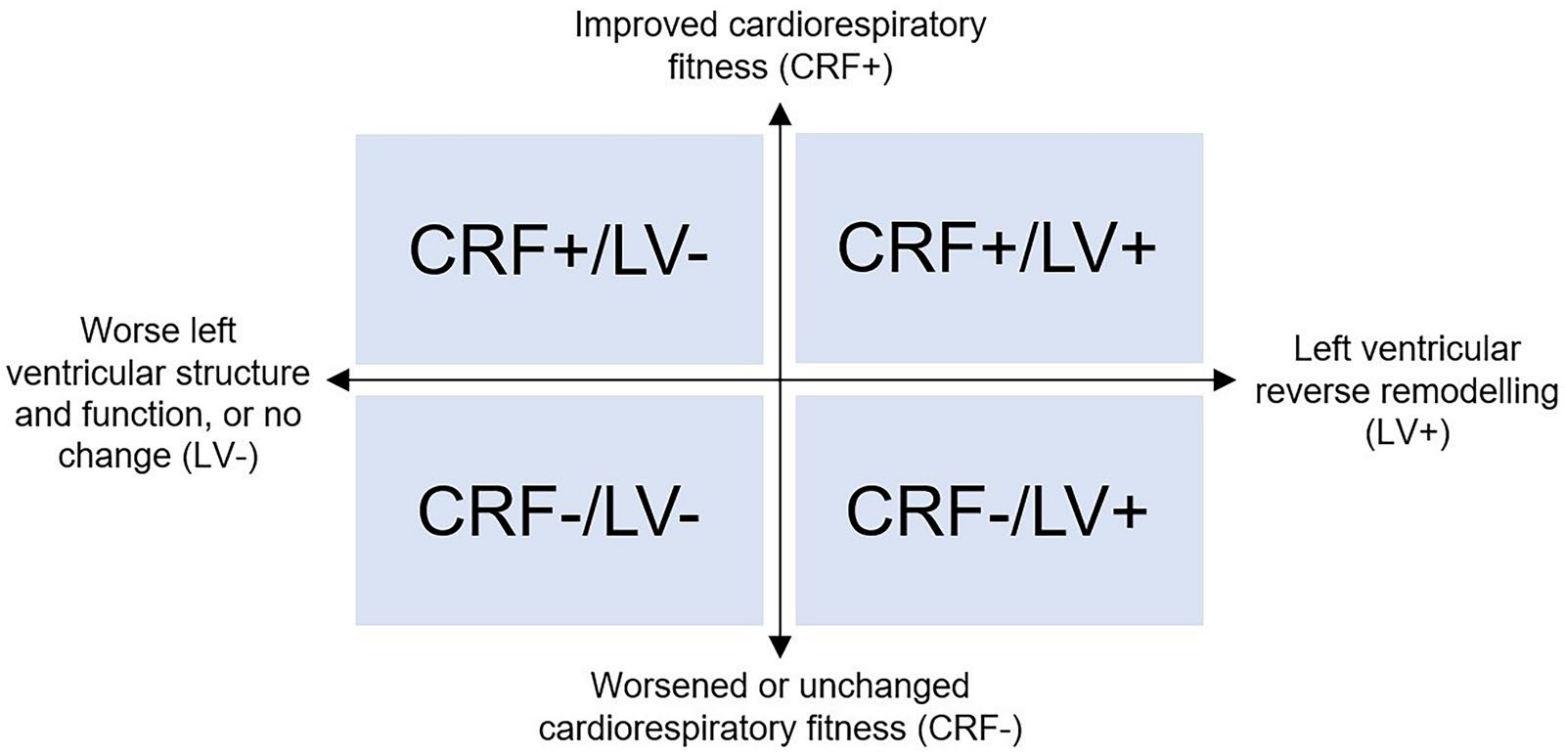
Possible recovery trajectories. *Abbreviations*: CRF+: Improved cardiorespiratory fitness; CRF-: Worsened or unchanged cardiorespiratory fitness; LV+: Left ventricular reverse remodeling; LV−: Worse left ventricular structure and function, or no change.

In conclusion, CRF tends to improve in KTRs after transplantation and is often better than CRF in dialysis patients. However, KTRs continue to perform below healthy controls, suggesting persistent limitations despite improved renal function. Improvements in fitness after transplant are often modest and vary across studies, likely due to differences in baseline fitness, post-transplant recovery time, individual physical activity levels and comorbidities. The remodeling of the left ventricle in KTRs does not appear to correspond to a significant improvement in cardiovascular reserve in this population, further highlighting the ‘transplant cardio-recovery gap’. This is demonstrated in Figure 1, which summarises the four possible recovery trajectories after transplant. The majority of studies are small, heterogeneous and have limited long-term data, highlighting the need for more robust, longitudinal research using CPET to fully characterise CRF in KTRs and determine whether this reduction in CRF represents and early plateau or a persistent deficit.

## The associations between cardiorespiratory fitness and cardiac structure and function in kidney transplant recipients: prognosis and phenotype stratification are critical

There is very limited research that directly compares CRF and cardiac structure and function in the KTR population. This evidence is summarised in Table 5.

**Table 5.** Study characteristics for studies exploring the relationship between cardiorespiratory fitness and cardiac structure and function KTRs.

Author year	Study type	Sample size	Follow up time	CPET type	Imaging modality	Summary of main outcomes
Bullock et al. 1984 [56]	Cross-sectional	54 patients on RRT including KT and dialysis	N/A	Cycle ergometer	Echo	Workload associated with cardiac abnormalities.
Ma et al. 2014 [58]	Cross-sectional	38 KTRs	N/A	Treadmill	Echo	Chronotropic incompetence associated with increased LVM, septal wall thickness and posterior wall thickness.
Lim et al. 2020 [44]	Prospective cohort	81 KTRs 85 CKD stage 5 87 hypertensive controls	1 year	Cycle ergometer	Echo	Increase in V̇O_2 peak,_ V̇O_2_AT and LVEF but no change in LVMi compared to baseline values.
Armstrong et al. 2006 [54]	Cross-sectional	71 KTRs 25 healthy age- and sex-matched controls	N/A	Treadmill	Echo	V̇O_2 peak_ associated with LVMi, LVEDV and LVESV.
Hellman et al. 2022 [57]	Prospective cohort	43 KTRs 50 CKD stage 4–5 on dialysis 29 CKD stage 4–5 not on dialysis	Within 3 years	Cycle ergometer	Echo	Mean workload in the last 4 min associated with LVEDD.

*Abbreviations:* KTR: kidney transplant recipient; KT: kidney transplant; RRT: renal replacement therapy; V̇O_2 peak_: peak oxygen uptake; V̇O_2_AT: oxygen uptake at the anaerobic threshold; LVMi: left ventricular mass index; LVEDV: left ventricular end-diastolic volume; LVESV: left ventricular end-systolic volume; LVEDD: left ventricular end-diastolic diameter.

In 1984, Bullock et al. conducted a cross-sectional study of 54 patients 27 months into renal replacement therapy including dialysis or kidney transplantation, and 62 healthy controls [56]. Exercise tolerance was determined using a cycle ergometer exercise test, where workload, blood pressure and an ECG were measured, and cardiac abnormalities were detected using ECG and echocardiography. Using the CPET data, participants were categorised into five groups based on their exercise ability. A significant inverse association was found between participants’ level of ability and the prevalence of cardiac abnormalities (*p* < 0.001), with 11% of those with the highest ability having abnormalities, compared to 42% with the lowest. However, as this study did not measure V̇O_2 peak_, its assessment of exercise capacity is significantly less accurate than modern studies. Additionally, it did not differentiate between those on dialysis and those who had received a kidney transplant, although the authors reported that there was no difference in exercise tolerance between the two groups.

In 2006, Armstrong et al. conducted a cross-sectional study of 71 glucose-intolerant KTRs at least one year post-transplant [54]. They found significant correlations between V̇O_2 peak_ and LVMi (*p* = 0.01), LVEDV (*p* = 0.004), and LVESV (*p* = 0.02). However, the study’s small sample size and focus on glucose-intolerant KTRs limit its generalisability to the broader KTR population. Additionally, a 2022 prospective cohort study of 122 participants exploring workload in KTRs found a correlation between mean workload in the last four minutes of maximal stress and LVEDD (*p* = 0.076, based on a *p* < 0.10 significance level) [57].

A 2014 cross-sectional study by Ma et al. examined chronotropic incompetence in 38 KTRs [58]. The study found that chronotropic incompetence was associated with increased LVM (216.5 ± 56.1 vs. 183.1 ± 40.0 g, *p* = 0.04), septal wall thickness (11.7 ± 1.4 vs. 10.7 ± 1.1 mm, *p* = 0.03), and posterior wall thickness (10.9 ± 1.9 vs. 9.5 ± 1.7 mm, *p* = 0.02). The association with LVM remained significant after adjustment for age, sex, duration of transplant, hypertension and use of beta-blockers (odds ratio: 1.03, *p* = 0.03). However, its small sample size and exclusion of participants with preexisting CVD or cardiovascular symptoms (e.g., chest pain, exertional dyspnea, and orthopnoea) limit its generalisability. Additionally, the majority of participants were on beta blockers, limiting the accuracy of the measurement of chronotropic incompetence.

In 2020, Lim et al. found an improvement in V̇O_2 peak_, V̇O_2_AT and LVEF in 81 KTRs 12 months after transplant, but no improvement in LVMi [44]. While no direct correlation was reported, the authors hypothesised that improved cardiac function without structural changes might be due to enhanced cardiovascular reserve.

Overall, this limited body of evidence suggests that poor CRF may be related to cardiac structure and function in KTRs. However, existing studies suffer from small sample sizes, methodological limitations, and reliance on echocardiographic over CMR imaging, despite the advantages of CMR.

Box 1 The transplant cardio-recovery gap: reverse remodeling does not equal functional resilience

**The Transplant Cardio-Recovery Gap: Why Reverse Remodeling Does Not Equal Functional Resilience**

**Definition:** Kidney transplantation frequently often leads to partial reverse remodeling of the left ventricle including reductions in LV mass, improvements in geometry, and improvements in functional markers such as LVEF and strain. However, these changes do not appear to reliably translate to improvements in CRF, as measured by CPET.

**Clinical significance:** Although patients may show structural and functional improvement on imaging, this does not necessarily result in improved fitness, leaving elevated cardiovascular risk.

*Abbreviations: LV* Left ventricular, *LVEF* Left ventricular ejection fraction, *CRF* Cardiorespiratory fitness, *CPET* Cardiopulmonary exercise testing

## Future directions

Persistent gaps remain between structural recovery and functional cardiovascular reserve, and the existing body of evidence is insufficient to address the ‘transplant cardio-recovery gap’. Addressing the ‘transplant cardio-recovery gap’ will require a multi-modal research approach encompassing mechanistic studies, advanced imaging, AI and adaptive rehabilitation trials.

## Mechanistic discovery research

Future research should explore the mechanisms that link transplantation, LV remodeling and CRF. Investigations may include biomarkers of myocardial stress and fibrosis, inflammation, endothelial function and skeletal muscle characteristics. This could facilitate our understanding of the poor functional outcomes in KTRs and will help inform therapeutic targets.

## CMR-CPET coupling studies

Longitudinal studies that combine CMR and CPET could be utilised to explore associations between LV functioning and CRF. Serial assessments of LV volumes, mass, strain and fibrosis alongside V̇O_2 peak_ and other CPET parameters could help develop understanding of how cardiac remodeling influences CRF in KTRs. This may help identify patients with structural cardiac recovery but persistently low CRF who may benefit from targeted rehabilitation, define thresholds for functional versus structure recovery, and provide data for AI-driven prediction models for patient-specific recovery trajectories.

## Artificial intelligence-guided CMR-CPET coupling models and digital twin phenotyping

Artificial intelligence offers the potential to integrate multi-modal, longitudinal data into dynamic prediction models. By combining repeated CMR imaging, CPET metrics, biomarkers and demographics, AI could be used to generate patient-specific ‘digital twins’ that simulate cardiovascular responses to interventions. This approach could be used to personalise rehabilitation by predicting which specific exercise modalities and intensities patients might benefit from.

## Artificial intelligence-driven phenotype clustering

Artificial intelligence models based on imaging, functional, biomarker and demographic data may also help identify clinically meaningful phenotypes of KTRs with similar structural and functional recovery trajectories. Defining such phenotypes would allow precision rehabilitation strategies and risk stratification, with different phenotypes potentially benefiting from different rehabilitation modalities.

## Adaptive transplant cardiac rehabilitation trials

In order to translate these ideas into practice, we would like to highlight a conceptual clinical trial framework: ‘The Adaptive Cardio-Transplant Fitness Trial Platform (ACT-FT)’. The proposed platform could stratify KTRs by baseline phenotypic cluster and explore the effects of various rehabilitation modalities including high intensity interval training, resistance training, neuromuscular electrical stimulation, or a combination. An adaptive design would allow for adjustment of the intensity and frequency of interventions based on real-time progress using interim CMR and CPET data in order to quantify the effectiveness of precision rehabilitation as well as potentially validate AI-driven models and digital twin predictions.

## Conclusion

It is clear that pathological changes in cardiac structure and function as well as poor CRF are common in KTRs compared with the general population. While they generally show improvement following transplantation or compared to ESKD patients on other forms of renal replacement, rates of cardiac events and cardiac mortality remain high. We have highlighted a ‘transplant cardio-recovery gap’ which describes the gap between cardiovascular remodeling and significant improvement in CRF post-transplant. To advance understanding of the ‘transplant cardio-recovery gap’, there is an urgent need for coordinated data registries integrating CMR, CPET, and clinical outcomes in KTRs to enable phenotyping, facilitate AI-driven modeling, and accelerate the development of precision rehabilitation strategies, with the aim of reducing the significant rates of cardiac morbidity and mortality in this patient group.

## Data Availability

Data sharing is not applicable to this article as no new data were created or analyzed in this study.
